# Long-term safety and efficacy of bimekizumab in axial spondyloarthritis: 2-year results from two phase 3 studies

**DOI:** 10.1093/rheumatology/keaf009

**Published:** 2025-01-11

**Authors:** Xenofon Baraliakos, Atul Deodhar, Désirée van der Heijde, Filip Van den Bosch, Marina Magrey, Walter P Maksymowych, Tetsuya Tomita, Huji Xu, Ute Massow, Tom Vaux, Chetan Prajapati, Myriam Manente, Alexander Marten, Lianne S Gensler

**Affiliations:** Rheumazentrum Ruhrgebiet Herne, Ruhr-University Bochum, Germany; Division of Arthritis and Rheumatic Diseases, Oregon Health & Science University, Portland, OR, USA; Department of Rheumatology, Leiden University Medical Center, Leiden, The Netherlands; Department of Internal Medicine and Pediatrics, Ghent University and VIB Center for Inflammation Research, Ghent, Belgium; Case Western Reserve University, University Hospitals, Cleveland, OH, USA; Department of Medicine, University of Alberta, Edmonton, AB, Canada; Graduate School of Health Science, Morinomiya University of Medical Science, Osaka, Japan; Department of Rheumatology and Immunology, Shanghai Changzheng Hospital, Affiliated to Second Military Medical University, Shanghai, People's Republic of China; UCB, Monheim am Rhein, Germany; UCB, Slough, UK; UCB, Slough, UK; UCB, Braine-l’Alleud, Belgium; UCB, Monheim am Rhein, Germany; Department of Medicine/Rheumatology, University of California, San Francisco, CA, USA

**Keywords:** spondyloarthritis, biological therapies, clinical trials and methods, cytokines and inflammatory mediators, autoinflammatory conditions

## Abstract

**Objectives:**

Bimekizumab, a monoclonal IgG1 antibody that selectively inhibits IL-17F in addition to IL-17A, previously demonstrated efficacy and was well tolerated to 1 year in patients with non-radiographic (nr-) and radiographic (r-) axial spondyloarthritis (axSpA). Here, we report bimekizumab safety and efficacy to 2 years.

**Methods:**

Patients completing week 52 in the phase 3 studies BE MOBILE 1 (nr-axSpA; NCT03928704) and 2 (r-axSpA; NCT03928743) were eligible for an ongoing open-label extension (OLE; NCT04436640). All OLE patients received subcutaneous bimekizumab 160 mg every 4 weeks. Safety outcomes for patients who received ≥1 bimekizumab dose, and efficacy outcomes for all randomized patients, are reported to week 104.

**Results:**

In the OLE (weeks 52 − 104), 70.8% (367/518) of patients reported ≥1 treatment-emergent adverse event (TEAE). Most frequent TEAEs [exposure-adjusted incidence rate per 100 patient-years (EAIR/100PY)] were SARS-CoV-2 (COVID-19) infection (25.2), nasopharyngitis (11.0) and oral candidiasis (5.4). Fungal infection EAIR/100PY was 11.8 (majority *Candida* infections: 6.8; most mild/moderate, none serious/systemic). Inflammatory bowel disease and uveitis rates were low; no major adverse cardiovascular events or deaths occurred. TEAE incidence rate was generally similar across weeks 0 – 52 and 52 – 104.

At week 104, >50% of randomized patients (*N* = 586) achieved Assessment of SpondyloArthritis international Society 40% response (ASAS40); ∼60% achieved Axial Spondyloarthritis Disease Activity Score (ASDAS) low disease activity (<2.1) and >30% achieved ASDAS inactive disease (<1.3). Bimekizumab demonstrated sustained suppression of MRI inflammation at week 104, with >57% of patients achieving MRI remission.

**Conclusions:**

The safety profile of bimekizumab remained consistent with prior reports, with no new safety signals identified. 1-year efficacy was sustained to 2 years across patients with nr-axSpA and r-axSpA.

## Graphical Abstract

**Figure:**
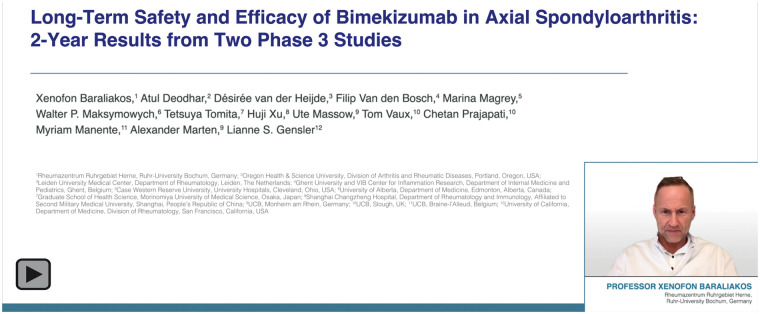

Rheumatology key messages

- Bimekizumab was well tolerated over 2 years in patients with axial spondyloarthritis (axSpA).
- Improvements in clinical outcomes were also sustained through 2 years in patients with axSpA.
- These results support the use of bimekizumab as a treatment option for patients with axSpA.

## Introduction

Axial spondyloarthritis (axSpA) is a chronic, immune-mediated inflammatory disease affecting the sacroiliac joints (SIJ) and spine (i.e. axial skeleton) [1]. Historically split into radiographic (r-)axSpA (i.e. ankylosing spondylitis) [2], in which structural damage is visible on plain radiographs, and non-radiographic (nr-)axSpA, in which patients lack such damage [1], axSpA is now recognized as a continuum of one disease [2–5]. Patients with nr-axSpA and r-axSpA share similar clinical presentations and disease burden [3, 6], facing significant impacts on physical functioning and health-related quality of life (HRQoL) [7] due to chronic inflammatory back pain, stiffness and extra-articular manifestations, including enthesitis and peripheral arthritis [1].

For patients with axSpA who do not respond to conventional first-line treatments [e.g. non-steroidal anti-inflammatory drugs (NSAIDs)], guidelines recommend targeted disease-modifying antirheumatic drugs (DMARDs), including tumour necrosis factor (TNF), interleukin (IL)-17 and Janus kinase (JAK) inhibitors [3, 8]. However, some DMARD-treated patients experience adverse events (AEs) or inadequate response [9–11], emphasizing the need for investigation of alternative treatments.

The cytokines IL-17A and IL-17F are key inflammatory mediators and have ∼50% structural homology, forming heterodimers and homodimers which signal through the IL-17 receptor complex [12]. While overexpression of IL-17A and IL-17F is connected to axSpA pathology [12], approved IL-17 inhibitors (e.g. secukinumab and ixekizumab) have, until recently, targeted IL-17A alone [3]. Multiple phase 3 studies have demonstrated the clinical efficacy of these axSpA treatments *vs* placebo [13–19], but pre-clinical studies suggest that dual blockade of IL-17A and IL-17F may be more efficacious in targeting axSpA pathology than IL-17A-only inhibition [12, 20, 21].

Bimekizumab, a monoclonal IgG1 antibody that selectively inhibits IL-17F in addition to IL-17A, was recently approved by the European Medicines Agency (EMA) and the Food and Drug Administration (FDA) to treat axSpA [22, 23]. The parallel phase 3 studies BE MOBILE 1 (NCT03928704) and BE MOBILE 2 (NCT03928743) demonstrated the safety and efficacy of bimekizumab to 1 year in patients with nr-axSpA and r-axSpA, respectively [24, 25]. Bimekizumab has also shown 5-year safety and efficacy in the treatment of r-axSpA in a phase 2 b study [26]. Furthermore, bimekizumab has shown therapeutic potential across other inflammatory diseases, demonstrating superior clinical efficacy *vs* the IL-17A-only inhibitor secukinumab in treating psoriasis [27], and *vs* placebo in treating psoriatic arthritis and hidradenitis suppurativa [28–30].

Here, we present interim 2-year data from the BE MOBILE studies, and their combined ongoing open-label extension (OLE) study BE MOVING (NCT04436640), to assess the long-term safety and efficacy of bimekizumab in axSpA.

## Methods

### Study design and oversight

The study design, inclusion and exclusion criteria for the BE MOBILE studies have been described previously (Supplementary Fig. S1, available at *Rheumatology* online) [24, 25].

These studies enrolled adult patients with active axSpA, defined as Bath Ankylosing Spondylitis Disease Activity Index (BASDAI) ≥4 and spinal pain (BASDAI item 2) ≥4 [24]. Patients in BE MOBILE 1 had nr-axSpA meeting Assessment of SpondyloArthritis International Society (ASAS) classification criteria and had objective inflammation at screening [active sacroiliitis on MRI and/or elevated C-reactive protein (CRP) ≥6.0 mg/l] [24]. Patients in BE MOBILE 2 had r-axSpA and fulfilled modified New York (mNY) criteria. All patients in BE MOBILE 2 also fulfilled ASAS criteria [24]. Both studies comprised a 16-week double-blind placebo-controlled period followed by a 36-week maintenance period [24, 25]. From week 16, all patients received subcutaneous bimekizumab 160 mg every 4 weeks (Q4W) to week 52.

At week 52, all patients who completed either study without meeting withdrawal criteria were eligible to enrol in the OLE, where all patients continued to receive bimekizumab 160 mg Q4W for a further 112 weeks (Supplementary Fig. S1, available at *Rheumatology* online). During the OLE treatment period, patients completed study assessments every 4 weeks to week 68 (i.e. OLE week 16), then every 12 weeks thereafter. Bimekizumab was administered subcutaneously via a 1 ml prefilled syringe by study personnel during the pre-specified assessment visits and via self-administration for interim visits from week 68 onwards. A safety follow-up visit was completed 20 weeks after the final bimekizumab dose (Supplementary Methods).

All study timepoints are reported relative to baseline (i.e. week 0) of the initial randomized BE MOBILE studies. Here, we report results up to week 104 (up to 2 years of total treatment duration).

### Study endpoints

The primary safety variables in the OLE were the incidence of treatment-emergent AEs (TEAEs), serious TEAEs and TEAEs leading to withdrawal from bimekizumab. TEAEs were defined as any AE occurring during treatment exposure, up to 140 days from the last treatment dose. TEAEs were classified using the Medical Dictionary for Regulatory Activities (MedDRA) version (v)19.0. Specific terms for SARS-CoV-2 (COVID-19) infections were not available in the MedDRA v19.0; confirmed or suspected cases were identified by the preferred terms ‘Corona virus infection’ and ‘Coronavirus test positive’.

SAEs were any AEs leading to ≥1 of: death, life-threatening event, significant or persistent disability/incapacity, congenital anomaly/birth defect (including in a foetus), important medical event, or initial inpatient hospitalization or prolonged hospitalization. Pre-defined cardiovascular, gastrointestinal and neuropsychiatric TEAEs were reviewed and adjudicated by independent Cardiovascular, Inflammatory Bowel Disease (IBD) and Neuropsychiatric Adjudication Committees, respectively. Suicidal ideation and behaviour were assessed by trained study personnel using the electronic Columbia-Suicide Severity Rating Scale (eC-SSRS). Pre-specified safety topics of interest were also assessed (Supplementary Methods).

Secondary efficacy variables included the proportion of patients achieving ASAS 40% response (ASAS40), ASAS20, ASAS partial remission (PR), ASAS 5/6, and change from baseline (CfB) in BASDAI, Axial Spondyloarthritis Disease Activity Score (ASDAS), and Bath Ankylosing Spondylitis Functional Index (BASFI). HRQoL measures included Ankylosing Spondylitis Quality of Life (ASQoL) and Short-Form 36-Item Health Survey Physical Component Summary (SF-36 PCS). Enthesitis and peripheral arthritis outcomes were measured using the Maastricht Ankylosing Spondylitis Enthesitis Score (MASES) and swollen joint count (SJC), respectively. Supplementary Table S1, available at *Rheumatology* online, contains a full list of secondary efficacy variables.

Pre-specified efficacy endpoints included MRI inflammation assessment. CfB in the Berlin modification of the spine ASspiMRI-a score (‘MRI Berlin spine score’) and MRI Spondyloarthritis Research Consortium of Canada (SPARCC) [31] SIJ inflammation score were measured in MRI sub-study patients. In the BE MOBILE studies, MRIs were performed on both the SIJ and spine of all patients with nr-axSpA and r-axSpA enrolled in the respective MRI sub-studies at baseline, week 16 and week 52. In the OLE, MRIs were performed at week 104 on the SIJ in patients with nr-axSpA and the spine in patients with r-axSpA only. MRIs were assessed by two central readers, with discrepancies adjudicated by a third reader (Supplementary Methods). The average of the two closest change scores was recorded. All readers were blinded to timepoint and clinical data. Proportions of patients achieving MRI remission, defined as MRI SPARCC SIJ score <2 (nr-axSpA) and MRI Berlin spine score ≤2 (r-axSpA), are reported for patients with respective baseline scores ≥2 and >2.

BASDAI question (Q)1 fatigue was not a pre-specified end point but is reported here as it is part of the ASAS-Outcome Measures in Rheumatology (OMERACT) core outcome set for axSpA [32].

### Statistical analysis

For these interim analyses, summary safety and efficacy data are reported to week 104, pooled across patients with nr-axSpA and r-axSpA.

All safety variables are summarized for all patients who received ≥1 bimekizumab dose (safety set). Exposure-adjusted incidence rates (EAIRs) of TEAEs per 100 patient-years (PY) are presented for weeks 0 – 52 (during the BE MOBILE studies) and 52 – 104 (during the OLE).

Analyses of secondary and other pre-specified efficacy variables were performed on all patients randomized in the BE MOBILE studies (randomized set). Efficacy data up to week 52, split by patients randomized to bimekizumab or placebo, have been published previously [24, 25]. From week 52, all data were pooled, regardless of initial randomized treatment arm. For efficacy measurements, CfB and response outcomes were analyzed relative to the baseline mean (week 0 of the BE MOBILE studies). These measurements were pooled across the full disease spectrum of axSpA, with the exception of MRI data which are reported split by nr-axSpA and r-axSpA, including between weeks 0 – 52. Statistical analysis methodology details for the BE MOBILE trials to week 52 have been reported previously [24, 25].

Missing data were generally imputed as non-responder imputation (NRI) for binary efficacy variables and as multiple imputation (MI) for analysis of continuous efficacy variables or continuous components of binary variables. Patients who did not enter the OLE were imputed as non-responders. Supportive observed case (OC) data are provided. Only OC score data are reported for CfB in MRI Berlin spine and MRI SPARCC SIJ inflammation scores. All statistical analyses were conducted in Statistical Analysis System (SAS; v9.3 or later).

## Results

### Patient disposition and baseline characteristics

Of the 586 patients randomized in the BE MOBILE studies (nr-axSpA: 254; r-axSpA: 332), 518 (88.4%) patients (nr-axSpA: 220; r-axSpA: 298) completed week 52 and 494 (84.3%) patients (nr-axSpA: 208; r-axSpA: 286) entered the OLE. From weeks 52 − 104, 20 patients with nr-axSpA and 23 patients with r-axSpA discontinued the study; most discontinuations were due to withdrawal of consent (Supplementary Fig. S2, available at *Rheumatology* online). By 6 July 2023, 456 (77.8%) patients (nr-axSpA: 189; r-axSpA: 267) had completed week 104 (Supplementary Fig. S2, available at *Rheumatology* online).

Demographics and baseline characteristics in the BE MOBILE studies were generally similar between treatment groups and across all patients with nr-axSpA and r-axSpA (Table 1).

**Table 1.** Patient demographics and baseline characteristics

	BE MOBILE 1 (nr-axSpA)		BE MOBILE 2 (r-axSpA)	
Mean (SD), unless otherwise stated	PBO *n* = 126	BKZ 160 mg Q4W *n* = 128	PBO *n* = 111	BKZ 160 mg Q4W *n* = 221
**Patient demographics**				
Sex, male, *n* (%)	65 (51.6)	73 (57.0)	80 (72.1)	160 (72.4)
Age, years	39.4 (11.8)	39.5 (11.1)	39.2 (12.6)	41.0 (12.1)
Geographical region,^a^ *n* (%)				
Asia^b^	13 (10.3)	15 (11.7)	21 (18.9)	40 (18.1)
Eastern Europe^c^	71 (56.3)	73 (57.0)	55 (49.5)	108 (48.9)
Western Europe^d^	33 (26.2)	31 (24.2)	32 (28.8)	67 (30.3)
North America^e^	9 (7.1)	9 (7.0)	3 (2.7)	6 (2.7)
BMI, kg/m^2^	27.7 (5.5)	27.2 (6.0)	27.1 (5.8)	26.8 (5.7)
**Disease characteristics**				
Time since first symptoms of axSpA, years	9.0 (9.0)	9.1 (8.7)	11.9 (8.6)	14.2 (11.0)
Time since first diagnosis of axSpA, years	3.6 (5.4)	3.7 (6.2)	5.7 (6.9)	6.7 (8.3)
HLA-B27 positive, *n* (%)	94 (74.6)	103 (80.5)	93 (83.8)	191 (86.4)
ASDAS	3.7 (0.7)	3.7 (0.8)	3.7 (0.8)	3.7 (0.8)^f^
hs-CRP, mg/L, geometric mean (geometric CV, %)	5.0 (230.5)	4.6 (297.7)	6.7 (197.4)	6.5 (275.0)
BASDAI	6.7 (1.3)	6.9 (1.2)	6.5 (1.3)	6.5 (1.3)
PtGADA^g^	6.9 (1.9)	7.1 (1.9)	6.7 (1.8)	6.6 (2.0)^f^
Total spinal pain^g^	7.1 (1.6)	7.3 (1.5)	7.2 (1.2)	7.1 (1.6)
Nocturnal spinal pain	6.7 (2.1)	6.9 (2.0)	6.8 (1.8)	6.6 (1.9)
Morning stiffness (mean of BASDAI Q5&6)^g^	6.9 (1.6)	7.0 (1.8)	6.8 (1.6)	6.7 (1.9)
BASFI^g^	5.3 (2.3)	5.5 (2.2)	5.2 (2.0)	5.3 (2.2)
BASMI	3.0 (1.2)	2.9 (1.3)	3.8 (1.6)^h^	3.9 (1.6)^f^
ASQoL	9.4 (4.4)	9.5 (4.6)	8.5 (4.3)	9.0 (4.7)
SF-36 PCS	33.6 (8.7)	33.3 (8.3)	34.6 (8.7)	34.3 (8.4)^f^
MRI Berlin spine score^i^	1.6 (2.9)^j^	1.6 (2.6)^k^	3.2 (4.1)^l^	3.3 (4.5)^m^
MRI SPARCC SIJ score^i^	9.8 (12.6)^n^	8.0 (9.9)^o^	3.8 (6.1)^l^	5.4 (8.4)^p^
Current enthesitis (MASES >0), *n* (%)	92 (73.0)	94 (73.4)	67 (60.4)	132 (59.7)
MASES,^q^ mean (SE)	4.9 (0.4)^r^	4.8 (0.3)^s^	4.4 (0.3)^t^	4.2 (0.3)^u^
Current peripheral arthritis (SJC >0), *n* (%)	43 (34.1)	45 (35.2)	22 (19.8)	44 (19.9)
History of IBD,^v^ *n* (%)	1 (0.8)	3 (2.3)	1 (0.9)	3 (1.4)
History of uveitis,^v^ *n* (%)	21 (16.7)	19 (14.8)	24 (21.6)	33 (14.9)
History of psoriasis,^v^ *n* (%)	7 (5.6)	9 (7.0)	10 (9.0)	16 (7.2)
Prior TNFi exposure (TNFi-IR patients),^w^ *n* (%)	17 (13.5)	10 (7.8)	17 (15.3)	37 (16.7)
Concomitant medication use at baseline, *n* (%)				
NSAIDs	93 (73.8)	96 (75.0)	85 (76.6)	181 (81.9)
Oral glucocorticoids	14 (11.1)	7 (5.5)	8 (7.2)	15 (6.8)
csDMARDs^x^	32 (25.4)	29 (22.7)	19 (17.1)	47 (21.3)

Randomized set. Patients in BE MOBILE 1 met ASAS criteria and patients in BE MOBILE 2 met mNY and ASAS criteria.

a Patients categorized by the stratum to which they were randomized.

b Includes China, Japan and Turkey.

c Includes Bulgaria, Czech Republic, Hungary and Poland.

d Includes Belgium, France, Germany, The Netherlands, Spain and United Kingdom.

e Includes United States of America only.

f *n* = 220.

g Individual component of the primary outcome measure (ASAS40).

h *n* = 108.

i In patients in the MRI sub-study.

j *n* = 67.

k *n* = 79.

l *n* = 48.

m *n* = 89.

n *n* = 70.

o *n* = 82.

p *n* = 90.

q In patients with MASES >0 at baseline.

r *n* = 92.

s *n* = 94.

t *n* = 67.

u *n* = 132.

v Based on extra-articular assessments at screening or baseline.

w Defined as patients who were intolerant or experienced an inadequate response to previous TNFi treatment given at an approved dose for at least 12 weeks.

x Includes methotrexate in 21 patients with nr-axSpA and 12 patients with r-axSpA, sulfasalazine in 33 patients with nr-axSpA and 52 patients with r-axSpA.

Abbreviations: ASAS: Assessment of SpondyloArthritis international Society; ASDAS: Axial Spondyloarthritis Disease Activity Score; ASQoL: Ankylosing Spondylitis Quality of Life; axSpA: axial spondyloarthritis; BASDAI: Bath Ankylosing Spondylitis Disease Activity Index; BASFI: Bath Ankylosing Spondylitis Functional Index; BASMI: Bath Ankylosing Spondylitis Metrology Index; BKZ: bimekizumab; BMI: body mass index; csDMARD: conventional synthetic disease-modifying antirheumatic drug; CV: co-efficient of variation; HLA-B27: human leucocyte antigen-B27; hs-CRP: high-sensitivity C-reactive protein; IBD: inflammatory bowel disease; IR: inadequate response; MASES: Maastricht Ankylosing Spondylitis Enthesitis Score; mNY: modified New York; MRI: magnetic resonance imaging; nr-axSpA: non-radiographic axial spondyloarthritis; NSAID: non-steroidal anti-inflammatory drug; PBO: placebo; PtGADA: Patient’s Global Assessment of Disease Activity; Q: question; Q4W: every 4 weeks; r-axSpA: radiographic axial spondyloarthritis; SD: standard deviation; SE: standard error; SF-36 PCS: Short-Form 36-item Health Survey Physical Component Summary; SIJ: sacroiliac joint; SJC: swollen joint count; SPARCC: Spondyloarthritis Research Consortium of Canada; TNFi: tumour necrosis factor inhibitor.

### Safety

#### Overview

Safety data from the BE MOBILE studies (weeks 0 − 52) have been reported previously [24, 25]; summary data are provided in Table 2 and Supplementary Table S2, available at *Rheumatology* online. Rates of TEAEs during the OLE (weeks 52 − 104) were generally similar to, or numerically lower than, those reported during weeks 0 − 52.

**Table 2.** Safety overview to week 104, split by year

	Any BKZ 160 mg Q4W	
*n* (%) [EAIR/100 PY]	Weeks 0 – 52	Weeks 52 – 104
	N = 574^a^; 551.5 PY	N = 518; 477.9 PY
	(BE MOBILE 1&2)	(BE MOVING)
**Overview**		
Any TEAE	451 (78.6) [196.4]	367 (70.8) [150.6]
Severe TEAEs	23 (4.0) [4.3]	16 (3.1) [3.4]
TEAEs leading to study discontinuation	21 (3.7) [3.9]	8 (1.5) [1.7]
TEAEs leading to BKZ discontinuation	24 (4.2) [4.4]	8 (1.5) [1.7]
Drug-related TEAEs	226 (39.4) [57.3]	137 (26.4) [34.5]
Serious TEAEs^b^	32 (5.6) [5.9]	28 (5.4) [6.0]
Death	0	0
**Most frequently reported TEAEs by preferred term (≥5%)** ^c^		
SARS-CoV-2 (COVID-19) infection^d^	35 (6.1) [6.5]	108 (20.8) [25.2]
Nasopharyngitis	61 (10.6) [11.9]	50 (9.7) [11.0]
Oral candidiasis	42 (7.3) [8.0]	25 (4.8) [5.4]
Upper respiratory tract infection	47 (8.2) [9.0]	24 (4.6) [5.1]
Headache	33 (5.7) [6.2]	13 (2.5) [2.7]
Diarrhoea	30 (5.2) [5.6]	9 (1.7) [1.9]
**Safety topics of interest**		
Serious infections	10 (1.7) [1.8]	6 (1.2) [1.3]
Opportunistic infections	8 (1.4) [1.5]	4 (0.8) [0.8]
Active tuberculosis	0	0
Fungal infections	83 (14.5) [16.5]	53 (10.2) [11.8]
*Candida* infections	52 (9.1) [10.0]	31 (6.0) [6.8]
Fungal infections NEC	28 (4.9) [5.2]	22 (4.2) [4.7]
*Tinea* infections	9 (1.6) [1.6]	6 (1.2) [1.3]
Neutropenia	5 (0.9) [0.9]	4 (0.8) [0.8]
Hepatic events^e^^,f^	54 (9.4) [10.4]	22 (4.2) [4.7]
Liver function analyses	39 (6.8) [7.4]	19 (3.7) [4.1]
ALT/AST >3 × ULN	18 (3.1) [3.3]	11 (2.2) [2.3]
ALT/AST >5 × ULN	6 (1.0) [1.1]	5 (1.0) [1.1]
Hypersensitivity reactions^g^^,^^h^	64 (11.1) [12.4]	41 (7.9) [9.1]
Injection site reactions^i^	22 (3.8) [4.1]	4 (0.8) [0.8]
Adjudicated MACE	0	0
Malignancies^j^	2 (0.3) [0.4]	2 (0.4) [0.4]
Adjudicated suicidal ideation and behaviour	2 (0.3) [0.4]^k^	0
Depression	2 (0.3) [0.4]	3 (0.6) [0.6]
Adjudicated IBD^l^	5 (0.9) [0.9]	4 (0.8) [0.8]
With prior history	1 (12.5) [13.7]^m^	0^n^
Without prior history	4 (0.7) [0.7]^o^	4 (0.8) [0.9]^p^
Uveitis^q^	14 (2.4) [2.6]	6 (1.2) [1.3]
With prior history	10 (10.5) [11.4]^r^	6 (6.6) [7.2]^s^
Without prior history	4 (0.8) [0.9]^t^	0^u^

Safety set. Includes all data available up to the last week 104 visit; if patients discontinued before week 104, data are reported for up to 140 days from the last treatment dose, up to the data cut (July 2023). MedDRA (v19.0).

a 12 patients did not receive BKZ and are not included.

b Serious TEAEs met one or more of the following criteria: death, life-threatening event, significant or persistent disability/incapacity, congenital anomaly/birth defect (including in a foetus), important medical event, or initial inpatient hospitalization or prolonged hospitalization.

c In order of decreasing frequency in Year 2.

d Specific terms for SARS-CoV-2 (COVID-19) infections were not available in the MedDRA v19.0; confirmed or suspected cases were identified using the preferred terms ‘Corona virus infection’ and ‘Coronavirus test positive’.

e Most reported hepatic events were associated with non-serious abnormal liver function elevations; those that were markedly abnormal were associated with factors other than the study treatment.

f Includes events in the SMQ ‘Drug-related hepatic disorders—comprehensive search (SMQ)’, excluding the following two sub-SMQs: ‘Liver neoplasms, benign (incl cysts and polyps) (SMQ)’ and ‘Liver neoplasms, malignant and unspecified (SMQ)’.

g Hypersensitivity reactions identified via the MedDRA SMQ ‘Hypersensitivity (SMQ)’.

h Most cases were dermatitis and eczema; there were no anaphylactic reactions to BKZ and no hypersensitivity events were classified as serious TEAEs.

i Includes events in the high-level term ‘Injection site reactions’.

j One clear cell renal carcinoma event in BE MOBILE 1, one superficial spreading melanoma stage I event in BE MOBILE 2. One gastric adenocarcinoma event and one papillary thyroid cancer event in the OLE, all adjudicated as not related to study drug by the Investigator.

k Both patients had a history of psychiatric disorders and/or ongoing traumatic and stressful circumstances.

l Definite or probable adjudicated IBD reported per external adjudication committee.

m *n* = 8.

n *n* = 7.

o *n* = 566.

p *n* = 511.

q Includes the preferred terms ‘autoimmune uveitis’, ‘uveitis’, ‘iridocyclitis’ and ‘iritis’.

r *n* = 95.

s *n* = 91.

t *n* = 479.

u *n* = 427.

ALT: alanine aminotransferase; AST: aspartate aminotransferase; BKZ: bimekizumab; EAIR: exposure-adjusted incidence rate; IBD: inflammatory bowel disease; MACE: major adverse cardiovascular event; NEC: not elsewhere classified; PY: patient-years; Q4W: every 4 weeks; SMQ: standardized medical query; TEAE: treatment-emergent adverse event; ULN: upper limit of normal; v: version.

From weeks 52 − 104, 518 patients received ≥1 bimekizumab dose (total exposure: 477.9 PY); 367/518 (70.8%) patients had ≥1 TEAE (EAIR/100 PY: 150.6) and 28/518 (5.4%) had ≥1 serious TEAE (EAIR/100 PY: 6.0). TEAEs leading to study discontinuation occurred in 8/518 (1.5%) patients (EAIR/100 PY: 1.7). No deaths were reported in the BE MOBILE studies or OLE to week 104. From weeks 52 − 104, the most frequently reported TEAEs were COVID-19 infection (20.8%; EAIR/100 PY: 25.2; coinciding with the COVID-19 pandemic), nasopharyngitis (9.7%; EAIR/100 PY: 11.0) and oral candidiasis (4.8%; EAIR/100 PY: 5.4). A summary of key safety data from weeks 52 − 104 is provided in Table 2.

#### Safety topics of interest

From weeks 52 − 104, serious infections were reported in six patients (1.2%; EAIR/100 PY: 1.3; Table 2), with two cases of appendicitis, and one case each of cellulitis, pneumonia, post-operative wound infection and pilonidal cyst. None led to treatment discontinuation.

Fungal infections were experienced by 53/518 patients (10.2%; EAIR/100 PY: 11.8) from weeks 52 − 104 (Supplementary Table S2, available at *Rheumatology* online). During the OLE, the majority of fungal infections were mucocutaneous and mild-to-moderate. No cases of serious or systemic fungal infections were reported; one non-serious severe fungal infection was reported (oesophageal candidiasis; EAIR/100 PY: 0.2). Most patients experiencing fungal infection presented with *Candida* infections (31/53; EAIR/100 PY: 6.8; Table 2), with oral candidiasis being the most frequently reported preferred term (25/31). The remainder were fungal infections not elsewhere classified (NEC; 22/53) and *Tinea* infections (6/53; Table 2). The majority of fungal infections NEC comprised fungal skin infections and oral fungal infections (Supplementary Table S2, available at *Rheumatology* online). From weeks 52 − 104, two patients discontinued bimekizumab due to *Candida* infections (both moderate cases of oral candidiasis).

Among other pre-specified safety topics of interest, no major adverse cardiovascular events (MACE) or adjudicated suicidal ideation and behaviour were reported from weeks 52 − 104. There were no active tuberculosis cases in the BE MOBILE studies or the OLE. Rates of depression were low throughout the OLE (EAIR/100 PY: 0.6). Rates of opportunistic infections (EAIR/100 PY: 0.8), neutropenia (EAIR/100 PY: 0.8) and malignancies (EAIR/100 PY: 0.4) were also low from weeks 52 – 104 (Table 2). No cases of hypersensitivity reaction were classified as serious. Most patients (37/41) experiencing hypersensitivity reactions were classified under skin and subcutaneous tissue disorders, including the high-level terms ‘dermatitis and eczema’ and ‘rashes, eruptions and exanthems NEC’. No anaphylactic reactions occurred in the OLE. Injection site reactions were experienced by four (0.8%) patients (EAIR/100 PY: 0.8); all were mild.

Hepatic events occurred in 22/518 (4.2%; EAIR/100 PY: 4.7; Table 2) patients. All hepatic events were non-serious, and the majority were transient liver function test elevations or abnormalities; none led to permanent treatment discontinuation. There were no confirmed cases of Hy’s law.

#### Extra-musculoskeletal manifestations

From weeks 52 − 104, definite or probable adjudicated IBD was reported in 4/518 patients (0.8%; EAIR/100 PY: 0.8); all cases were in patients with no prior history of IBD at baseline. Uveitis events occurred in 6/518 patients (1.2%; EAIR/100 PY: 1.3); all cases occurred in patients with prior history of uveitis at baseline.

### Efficacy

A summary of all efficacy outcomes is provided in Table 3.

**Table 3.** Efficacy outcomes to week 104

		BE MOVING full randomized set		
		N = 586		
OC: *n*/N (%)		Baseline	Week 52	Week 104
MI: Mean % (95% CI)				
ASAS40	OC	—	347/516 (67.2)	304/456 (66.7)
	MI	—	62.4 (58.4, 66.4)	60.1 (55.9, 64.2)
ASAS20	OC	—	429/516 (83.1)	381/456 (83.6)
ASAS PR	OC	—	183/514 (35.6)	182/455 (40.0)
ASAS 5/6	OC	—	334/503 (66.4)	286/456 (62.7)
ASDAS MI	OC	—	204/499 (40.9)	207/449 (46.1)
	MI	—	37.0 (33.0, 40.9)	39.8 (35.7, 43.9)
ASDAS LDA (ASDAS <2.1)	OC	—	308/499 (61.7)	296/449 (65.9)
	MI	—	57.2 (53.1, 61.3)	59.7 (55.6, 63.8)
ASDAS ID (ASDAS <1.3)	OC	—	145/499 (29.1)	159/449 (35.4)
	MI	—	26.5 (22.9, 30.2)	30.6 (26.7, 34.5)
BASDAI50	OC	—	319/515 (61.9)	316/455 (69.5)
Total resolution of enthesitis (MASES = 0)^a^	OC	—	190/328 (57.9)	184/292 (63.0)
**MI: Mean (SE)**		**Mean at baseline**	**CfB to week 52**	**CfB to week 104**
ASDAS	MI	3.7 (0.0)	−1.8 (0.0)	−1.8 (0.0)
BASDAI	MI	6.6 (0.1)	−3.7 (0.1)	−3.9 (0.1)
BASFI	MI	5.3 (0.1)	−2.8 (0.1)	−2.9 (0.1)
BASMI	MI	3.5 (0.1)	−0.6 (0.0)	−0.6 (0.1)
MASES^a^	MI	4.5 (0.2)	−3.1 (0.2)	−3.2 (0.2)
Nocturnal spinal pain	MI	6.7 (0.1)	−4.2 (0.1)	−4.3 (0.1)
Morning stiffness (mean of BASDAI Q5 & Q6)	MI	6.8 (0.1)	−4.2 (0.1)	−4.3 (0.1)
Fatigue (BASDAI Q1)	MI	6.5 (0.1)	−3.1 (0.1)	−3.4 (0.1)
ASQoL	MI	9.1 (0.2)	−5.6 (0.2)	−5.6 (0.2)
SF-36 PCS	MI	34.0 (0.4)	12.0 (0.4)	12.4 (0.4)
PtGADA	MI	6.8 (0.1)	−3.9 (0.1)	−3.9 (0.1)
TJC^b^	MI	5.7 (0.3)	−4.0 (0.3)	−4.4 (0.3)
SJC^c^	MI	4.2 (0.4)	−3.2 (0.3)	−3.4 (0.3)

a MASES score reported in patients with MASES > 0 at baseline (*n* = 385).

b Assessed in patients with TJC > 0 at baseline (*n* = 340).

c Assessed in patients with SJC >0 at baseline (*n* = 154).

ASAS: Assessment of SpondyloArthritis international Society; ASAS20: ASAS 20% response; ASAS40: ASAS 40% response; ASAS 5/6: ASAS five out of six response criteria; ASAS PR: ASAS partial remission; ASDAS: Axial Spondyloarthritis Disease Activity Score; ASDAS ID: ASDAS inactive disease; ASDAS LDA: ASDAS low disease activity; ASDAS MI: ASDAS major improvement; ASQoL: Ankylosing Spondylitis Quality of Life; BASDAI: Bath Ankylosing Spondylitis Disease Activity Index; BASDAI50: BASDAI 50% response; BASFI: Bath Ankylosing Spondylitis Functional Index; BASMI: Bath Ankylosing Spondylitis Metrology Index; CfB: change from baseline; MASES: Maastricht Ankylosing Spondylitis Enthesitis Score; MI: multiple imputation; OC: observed case; PtGADA: Patient’s Global Assessment of Disease Activity; Q: question; SE: standard error; SF-36 PCS: Short-Form 36-Item Health Survey Physical Component Summary; SJC: swollen joint count; TJC: tender joint count.

#### Disease activity

**Figure 1.**
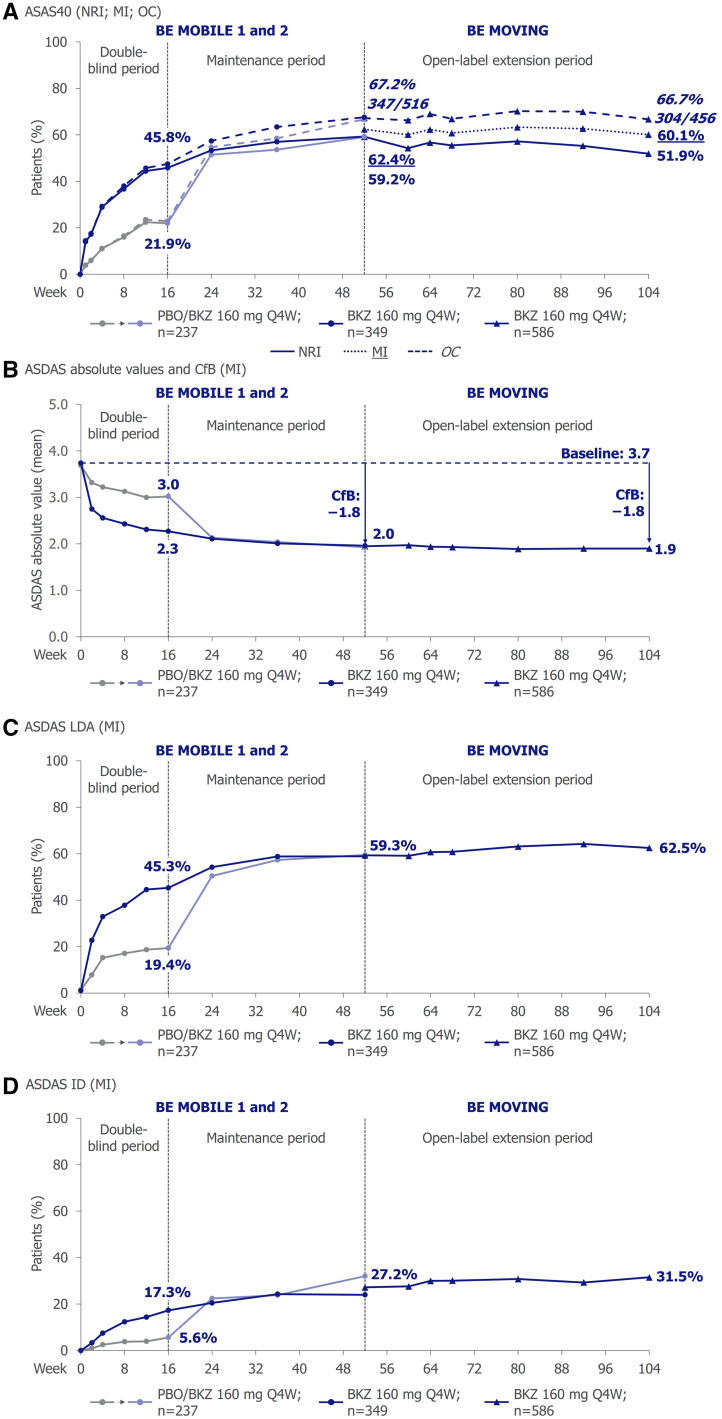
ASAS and ASDAS outcomes to week 104. Randomized set; all patients treated with BKZ 160 mg Q4W from week 16. Data reported using NRI and OC from week 0 – 104, in addition to MI from week 52 – 104. The dashed vertical line between weeks 48 and 56 indicates entry into the combined open-label treatment period (BE MOVING) following week 52 of BE MOBILE 1 or BE MOBILE 2. ASAS: Assessment of SpondyloArthritis international Society; ASAS40: ASAS 40% response; ASDAS: Axial Spondyloarthritis Disease Activity Score; ASDAS ID: ASDAS inactive disease; ASDAS LDA: ASDAS low disease activity; BKZ: bimekizumab; CfB: change from baseline; MI: multiple imputation; NRI: non-responder imputation; OC: observed case; PBO: placebo; Q4W: every 4 weeks.

At week 52, over half of patients achieved ASAS40, the primary outcome at week 16 in the BE MOBILE studies [24], including those who switched from placebo to bimekizumab at week 16 (NRI: 59.2%; OC: 67.2% [347/516]; MI: 62.4%). The proportion of patients achieving ASAS40 was sustained to week 104 (NRI: 51.9%; OC: 66.7% [304/456]; MI: 60.1%; Table 3; Fig. 1). Responder rates of other ASAS outcomes (e.g. ASAS20 and ASAS PR) at week 52 were generally maintained or further improved to week 104 (Table 3).

The mean reduction (i.e. improvement) from baseline in ASDAS score at week 52 [baseline score: 3.7; CfB: −1.8 (MI)] was sustained to week 104 [CfB: −1.8 (MI); Fig. 1; Table 3; Supplementary Tables S3–S5, available at *Rheumatology* online].

**Figure 2.**
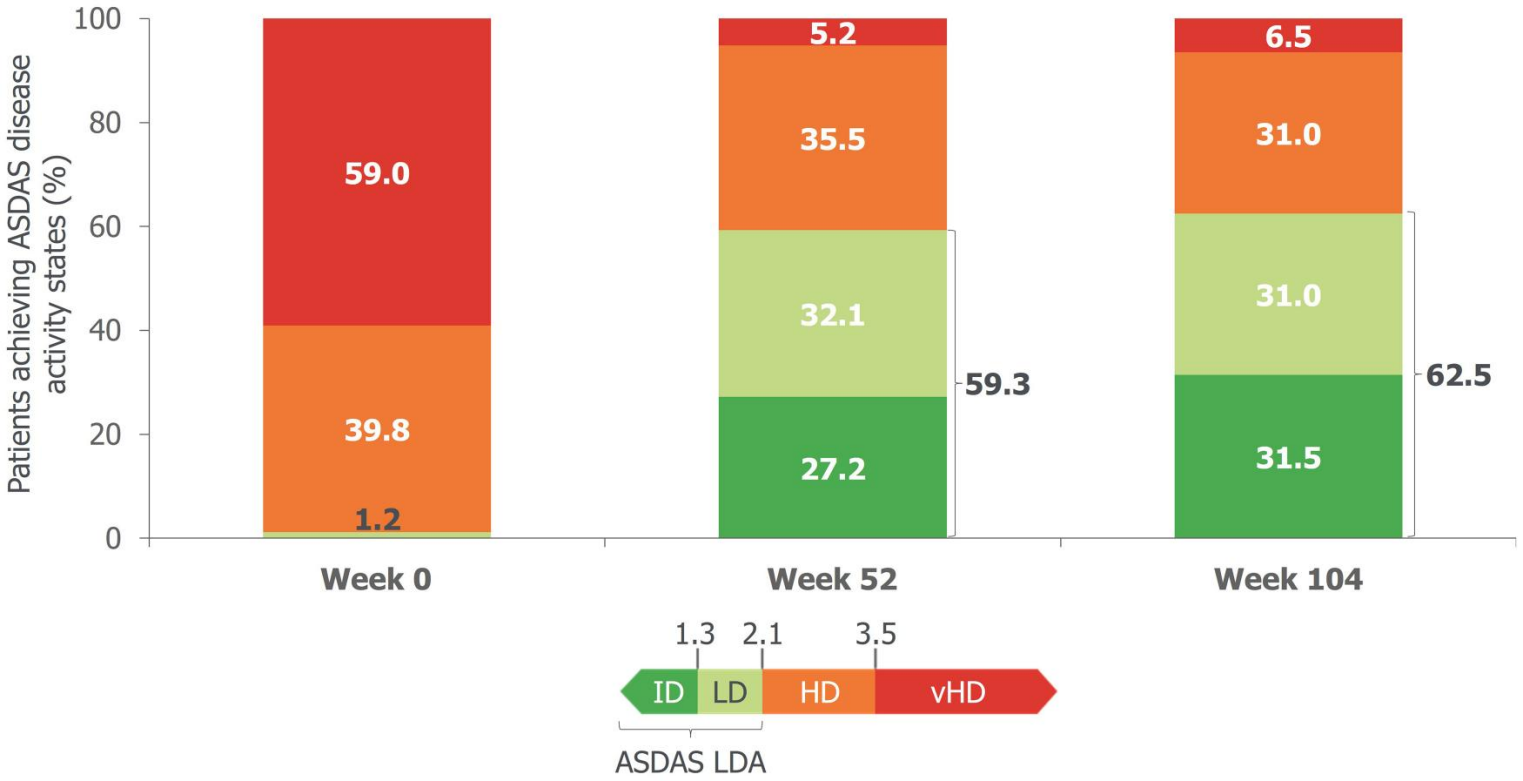
ASDAS disease states to week 104 (MI). *n* =586. Includes all patients, including those originally randomized to placebo, and any with intercurrent events; all patients were treated with BKZ 160 mg Q4W from week 16. Values shown to the right of the bars at week 52 and week 104 represent the proportion of patients achieving ASDAS LDA (that is, ASDAS <1.3 and ASDAS ≥1.3 to <2.1). ASDAS: Axial Spondyloarthritis Disease Activity Score; HD: high disease: ID: inactive disease; LD: low disease; LDA: low disease activity; MI: multiple imputation; vHD: very high disease.

At baseline, 98.8% of patients had ASDAS high/very high disease activity (ASDAS HDA/vHDA; i.e. ASDAS ≥2.1). At week 52, 59.3% achieved ASDAS low disease activity (ASDAS LDA; i.e. ASDAS <2.1) and 27.2% achieved ASDAS inactive disease (ASDAS ID; i.e. ASDAS <1.3). The proportion of patients achieving ASDAS LDA was sustained to week 104 (ASDAS LDA: 62.5%; ASDAS ID: 31.5%; Figs 1 and 2; Table 3). Similarly, the mean reduction (i.e. improvement) in total BASDAI score at week 52 [baseline score: 6.6; CfB: −3.7 (MI)] was also sustained to week 104 [CfB: −3.9 (MI); Table 3; Supplementary Tables S3–S5, available at *Rheumatology* online].

#### Pain, morning stiffness, fatigue and physical function

Improvements at week 52 in pain (i.e. nocturnal spinal pain), morning stiffness (i.e. mean BASDAI Q5&Q6), fatigue (i.e. BASDAI Q1) and physical function (i.e. BASFI; Table 3; Supplementary Tables S3–S5, available at *Rheumatology* online) were sustained to week 104.

#### Overall functioning and health

Improvements in HRQoL (i.e. ASQoL and SF-36 PCS) outcomes at week 52 were also sustained to week 104 (Table 3; Supplementary Tables S3–S5, available at *Rheumatology* online).

#### Peripheral manifestations

At baseline, 65.7% (385/586) of patients had enthesitis (MASES >0). Mean improvements in MASES at week 52 were sustained to week 104 (Table 3; Supplementary Tables S3–S5, available at *Rheumatology* online). At week 104, 184/385 (47.8%) patients with baseline MASES >0 achieved total resolution of enthesitis [MASES = 0 (NRI)].

For peripheral arthritis, at baseline, 26.3% (154/586) of patients had SJC >0. Improvements in SJC at week 52 were sustained to week 104 (Table 3; Supplementary Tables S3–S5, available at *Rheumatology* online) and, at week 104, 99/154 (64.3%) patients with baseline SJC >0 achieved total resolution of peripheral arthritis [SJC = 0 (NRI)].

#### Efficacy outcomes stratified by axSpA classification

The pattern of improvements in efficacy outcomes to week 52, which were sustained to week 104, were observed consistently across patients with nr-axSpA and r-axSpA (Supplementary Tables S3–S5, available at *Rheumatology* online).

#### MRI inflammation

**Figure 3.**
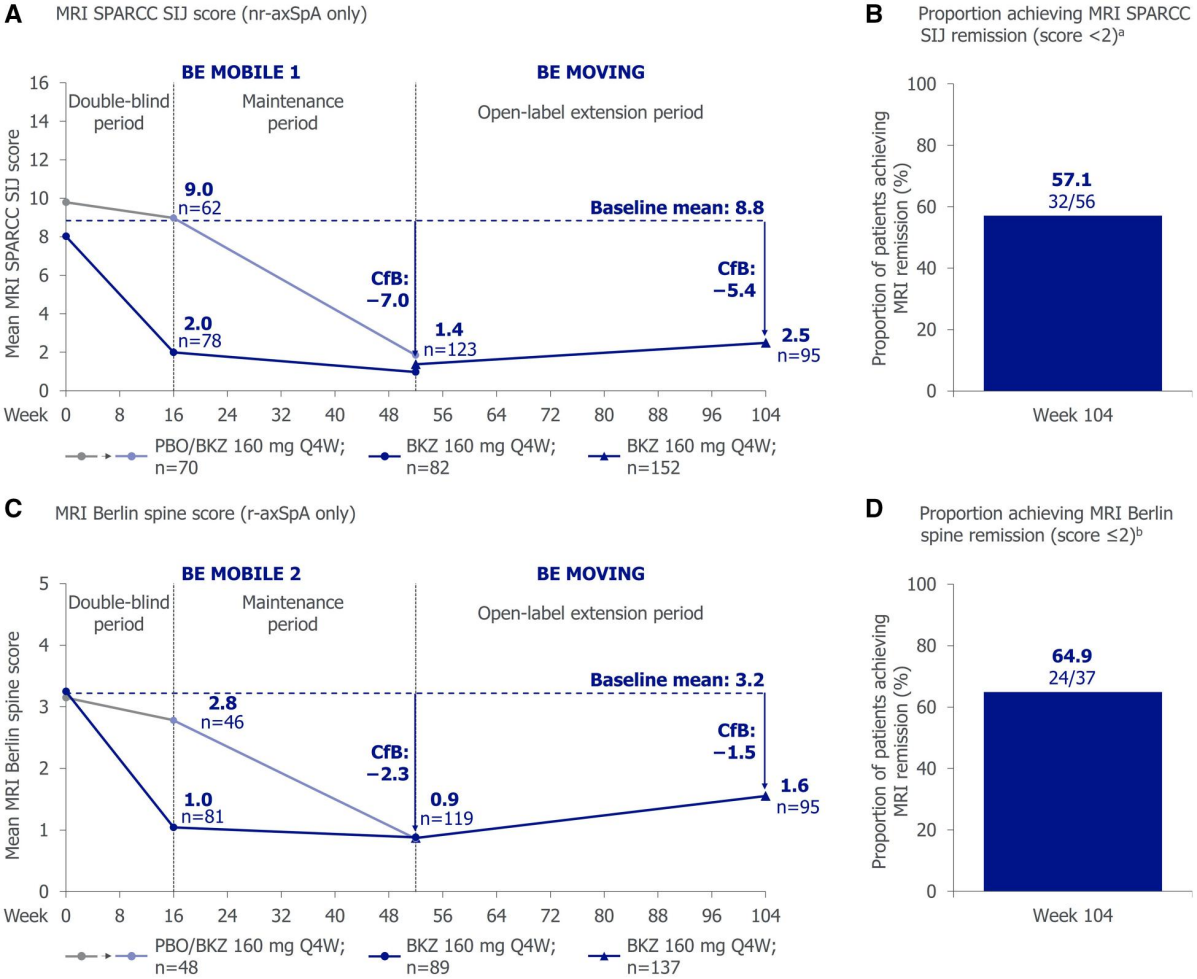
Absolute MRI inflammation scores and remission rates to week 104 (OC). Only study participants enrolled in the SIJ and spine MRI sub-study are included in this analysis. MRI remission is defined as having an MRI SPARCC SIJ score <2 or MRI Berlin spine score ≤2. The dashed vertical line between weeks 48 and 56 indicates entry into the combined open-label treatment period (BE MOVING) following week 52 of BE MOBILE 1 or BE MOBILE 2. ^a^Only sub-study participants with baseline MRI SPARCC SIJ score ≥2 included. ^b^Only sub-study participants with baseline MRI Berlin spine score >2 included. axSpA: axial spondyloarthritis; BKZ: bimekizumab: CfB: change from baseline; OC: observed case; MRI: magnetic resonance imaging; nr-axSpA: non-radiographic axSpA; PBO: placebo; Q4W: every 4 weeks; r-axSpA: radiographic axSpA; SD: standard deviation; SIJ: sacroiliac joint; SPARCC: Spondyloarthritis Research Consortium of Canada.

The reductions from baseline (i.e. improvements) in MRI inflammation observed with bimekizumab to week 52 in patients with nr-axSpA (MRI SPARCC SIJ) and r-axSpA (MRI Berlin spine) were sustained to week 104. At week 104, 57.1% (32/56) of patients with nr-axSpA achieved MRI remission (i.e. MRI SPARCC SIJ score <2), as did 64.9% (24/37) of patients with r-axSpA (i.e. MRI Berlin spine score ≤2; Fig. 3).

## Discussion

In the BE MOBILE studies and their OLE, dual inhibition of IL-17A and IL-17F with bimekizumab was well tolerated by patients across the full disease spectrum of axSpA. Improvements in efficacy outcomes at week 52 were largely sustained to week 104 and were consistent patients with nr-axSpA and r-axSpA.

In this ongoing OLE of two axSpA studies, no new safety signals were observed out to 2 years, and the long-term safety profile of bimekizumab was comparable to that previously established up to 5 years in the phase 2 b BE AGILE trial and its OLE in patients with r-axSpA [26], and also with other phase 3 trials of bimekizumab in psoriatic arthritis and psoriasis [27, 33]. There was also no increased risk of TEAEs with longer bimekizumab exposure. EAIRs for most safety topics of interest remained stable or numerically decreased in the second year, with the exception of COVID-19 infections, which were more common in Year 2 (coinciding with the global pandemic).

Throughout the OLE, the majority of fungal infections were mild-to-moderate, mucocutaneous *Candida* infections, such as oral candidiasis, likely due to the role of IL-17 in mucosal host defences against fungal infections (particularly those caused by *Candida*) [34, 35]. No cases of serious or systemic *Candida* infections were reported. Rates of other types of fungal infection, including *Tinea* infections, as well as serious and opportunistic infections, were low.

While IL-17F has been implicated in the pathophysiology of IBD, the underlying pathobiology of gastrointestinal TEAEs related to IL-17 inhibitor use is not yet fully understood [36, 37]. Throughout the study, EAIRs of definite or probable adjudicated IBD were low (week 52 EAIR/100 PY: 0.9; week 104 EAIR/100 PY: 0.8) and aligned with expected IBD rates in the axSpA population [38]. From weeks 52 – 104, all four cases of definite or probable adjudicated IBD occurred in patients without a prior history of IBD. This observation should be interpreted in the context of the study design, as the exclusion criteria precluded enrolment of patients with active symptomatic IBD [24].

The pathophysiology of uveitis, one of the most common extra-musculoskeletal manifestations of axSpA [1], is complex and multiple potential therapeutic targets including IL-17 and TNF-alpha have been identified [39]. Incidence of uveitis was low throughout the study and the six patients who experienced uveitis events during the OLE all had prior history of uveitis. Overall, rates of uveitis were comparable to pooled uveitis data from phase 2 b/3 bimekizumab trials across the full disease spectrum of axSpA [40].

Regarding efficacy, clinical outcome improvements achieved at 1 year were sustained out to 2 years across patients with nr-axSpA and r-axSpA. At week 104, ∼60% of patients achieved ASDAS LDA, a recommended key treatment target for axSpA [3, 41], and >30% of patients achieved ASDAS ID, a more stringent outcome representing remission [41]. High levels of improvement in the ASAS-OMERACT domains of pain, morning stiffness, fatigue and physical function were also sustained out to week 104. These results add to previous reports of rapid improvements in clinical efficacy outcomes to week 16 with bimekizumab and sustained efficacy from week 16 through 1 year [24, 25]. These results are also in line with the long-term efficacy results reported for patients with r-axSpA out to 5 years in the BE AGILE OLE [26], and the efficacy demonstrated with bimekizumab in other phase 3 trials in other indications [27].

Improvements in peripheral manifestations observed at week 52 were maintained throughout the OLE, with ∼50% of patients with MASES >0 at baseline achieving total resolution of enthesitis at week 104, as well as >60% of patients with baseline SJC >0 achieving total resolution of peripheral arthritis. Improvements in MRI inflammation achieved at week 52 were also maintained throughout the OLE, with >57% of patients achieving MRI remission at week 104.

In addition to the large sample size, a key strength of the BE MOBILE studies, and their OLE, is the inclusion of the full disease spectrum of axSpA. This manuscript is the first to report on the long-term 2-year safety and efficacy of bimekizumab across patients with nr-axSpA and r-axSpA. Data for missing efficacy data were reported using the most conservative imputation method (NRI), ensuring stringent interpretation of outcomes assessed throughout the study. Additionally, blinding all readers to timepoint when centrally assessing MRI imaging in the MRI sub-studies increased the objectivity of scan interpretation.

Limitations of the BE MOBILE studies and their OLE include a lack of active comparator arm. Therefore, direct comparisons between bimekizumab and other bDMARDs, such as IL-17A-only inhibitors, are not currently possible in axSpA. The BE MOBILE studies and their OLE also lacked a placebo arm after week 16, meaning patients were aware that they were receiving active treatment from week 16 onwards. Furthermore, ASQoL, a tool validated for both patients with nr-axSpA and r-axSpA [42–44] was used to assess HRQoL rather than the ASAS Health Index [45], the preferred measuring instrument according to the ASAS-OMERACT core outcome set [46]. MRI inflammation data were also limited by the smaller sub-study sample size and the diverging study protocol for MRI assessments of the spine and SIJ at week 104, with SIJ only assessed in patients with nr-axSpA and spine only assessed in patients with r-axSpA at this timepoint.

In conclusion, bimekizumab treatment demonstrated a favourable long-term safety profile over 2 years, consistent with observations from the BE MOBILE studies, previous phase 2 b studies in r-axSpA and phase 3 studies in other indications. Dual inhibition of IL-17A and IL-17F also resulted in sustained efficacy through 2 years, with improvements in disease activity, patient symptoms, HRQoL, physical function and MRI inflammation observed consistently across the full disease spectrum of axSpA. The consistent safety profile and long-term maintenance of clinical response provide robust evidence supporting bimekizumab as an important treatment option for patients with axSpA.

## Supplementary Material

keaf009_Supplementary_Data

## Data Availability

Data are available on reasonable request. Underlying data from this manuscript may be requested by qualified researchers six months after product approval in the US and/or Europe, or global development is discontinued, and 18 months after trial completion. Investigators may request access to anonymized individual patient-level data and redacted trial documents which may include: analysis-ready datasets, trial protocols, annotated case report forms, statistical analysis plans, dataset specifications and clinical study reports. Prior to use of the data, proposals need to be approved by an independent review panel at www.vivli.org and a signed data sharing agreement will need to be executed. All documents are available in English only, for a pre-specified time, typically 12 months, on a password-protected portal.
