# Supplementary material for: Long-term safety and efficacy of bimekizumab in axial spondyloarthritis: 2-year results from two phase 3 studies
Source: Rheumatology (Oxford). 2025 Jan 11;64(6):3534–46. doi: 10.1093/rheumatology/keaf009 (PMC12107048; doi:10.1093/rheumatology/keaf009)
Supplement: keaf009_Supplementary_Data [file keaf009_Supplementary_Data.pdf]

Long-Term Safety and Efficacy of Bimekizumab in Axial Spondyloarthritis: 2-Year Results from Two Phase 3 Studies

Xenofon Baraliakos,[1] Atul Deodhar,[2] Désirée van der Heijde,[3] Filip Van den Bosch,[4] Marina Magrey,[5] Walter P. Maksymowych,[6] Tetsuya Tomita,[7] Huji Xu,[8] Ute Massow,[9] Tom Vaux,[10] Chetan Prajapati,[10] Myriam Manente,[11] Alexander Marten,[9] Lianne S. Gensler[12]

1. *Rheumazentrum Ruhrgebiet Herne, Ruhr-University Bochum, Germany*
2. *Oregon Health & Science University, Division of Arthritis and Rheumatic Diseases, Portland, Oregon, USA*
3. *Leiden University Medical Center, Department of Rheumatology, Leiden, The Netherlands*
4. *Ghent University and VIB Center for Inflammation Research, Department of Internal Medicine and Pediatrics, Ghent, Belgium*
5. *Case Western Reserve University, University Hospitals, Cleveland, Ohio, USA*
6. *University of Alberta, Department of Medicine, Edmonton, Alberta, Canada*
7. *Graduate School of Health Science, Morinomiya University of Medical Science, Osaka, Japan*
8. *Shanghai Changzheng Hospital, Department of Rheumatology and Immunology, Affiliated to Second Military Medical University, Shanghai, People's Republic of China*
9. *UCB, Monheim am Rhein, Germany*
10. *UCB, Slough, UK*
11. *UCB, Braine-l'Alleud, Belgium*
12. *University of California, Department of Medicine/Rheumatology, San Francisco, California, USA*

Correspondence to: Prof. Baraliakos (Rheumazentrum Ruhrgebiet Herne, Ruhr-University Bochum, Germany; baraliakos@me.com)

Short title: BE MOVING 2-YR Safety and Efficacy Manuscript

Funding: UCB

Key words: Spondyloarthritis; Biological therapies; Clinical trials and methods; Cytokines and inflammatory mediators; Autoinflammatory conditions

SUPPLEMENTARY APPENDIX

SUPPLEMENTARY METHODS

Study design and oversight

Bimekizumab (BKZ) was administered subcutaneously via a 1 mL prefilled syringe by study personnel during the pre-specified assessment visits. Patients were given the option to self-administer at home at interim study visits (i.e., Weeks 20, 24, 32, 36, 44, 48, 56, 60, 68, 72, 80, 84, 92, 96, 104 and 108). No assessments were performed during home administrations. For patients where home administration was not possible, clinical site visits were permitted for treatment administration only.

Study endpoints

Pre-specified safety topics of interest for open-label extension (OLE) were infections (serious, opportunistic, fungal and tuberculosis), neutropenia, hypersensitivity, suicidal ideation and behaviour, depression, major adverse cardiovascular events (MACE), liver function test changes and enzyme elevations, malignancies and inflammatory bowel disease. Secondary endpoints and other pre-specified endpoints for efficacy outcomes can be found in **Table S1**.

MRI assessment methodology

MRI was used to visualise active inflammation in the sacroiliac joints (SIJ) and spine. MRI assessments were conducted for eligible patients who consented to the MRI sub-study of each trial. MRI Spondyloarthritis Research Consortium of Canada (SPARCC) SIJ scores range from 0–72, and MRI Berlin spine scores range from 0–69, with lower scores indicating less inflammation.

MRI sub-study methods have been reported previously.[25] In BE MOBILE 1 and 2, MRIs were performed on the SIJ and spine of all patients enrolled in the respective MRI sub-studies at baseline, Week 16 and Week 52. In the OLE, MRIs were performed at Week 104 on the SIJ in patients with nr-axSpA and the spine in patients with r-axSpA only.

All MRIs were read by two independent central readers; the analysis used the average of the change scores from these readers. Where discrepancies between readers were too large (MRI SPARCC SIJ inflammation: ≥13 difference in change score; MRI Berlin spine: ≥9 difference in change score), a third adjudication reviewer

was used. If adjudication was required, the average of the two closest change scores was recorded. All readers were blinded to timepoint and any clinical data.

SUPPLEMENTARY TABLES AND FIGURES

Table S1. Efficacy outcome measures to Week 104

	**Outcome measure**	**Notes**
**Secondary endpoints**	ASAS	• 4-domain score (PtGADA, pain assessment, function, inflammation), assessed using numeric rating scales from 0–10 in each domain<br>• ASAS20 – relative improvement of ≥20% and absolute improvement of ≥1 in ≥3 domains, with no deterioration in the remaining domain<br>• ASAS40 – relative improvement of ≥40% and absolute improvement of ≥1 in ≥3 domains, with no deterioration in the remaining domain<br>• ASAS PR – score of ≤2 in all 4 domains<br>• ASAS 5/6 – ≥20% improvement all 4 domains, in addition to 2 further domains measuring spinal mobility and CRP
	CfB in BASDAI	• Consists of 6 self-reported 10-unit numeric rating scales to measure severity of fatigue, spinal and peripheral joint pain and swelling, enthesitis, and morning stiffness over the last week<br>• Overall score ranges from 0–10<br>• Lower scores indicate lower disease activity
	CfB in ASDAS	• Comprised of assessments scored by both patients and Investigators, including total back pain, duration of morning stiffness, PtGADA, peripheral pain/swelling, and CRP<br>• Domains assessed on a numerical rating scale of 0–10, and summed to give a total score
	CfB in BASFI	• Validated axSpA-specific instrument for assessing physical function<br>• Comprises 10 score items relating to past week, ranging 0–10<br>• Lower scores indicate better physical function
	CfB in nocturnal spinal pain	• Assessed using a numeric rating scale from 0–10<br>• Higher scores indicate more severe pain
	CfB in ASQoL	• 18-item HRQoL measure, with each item scoring 1 (‘Yes’) or 0 (‘No’)<br>• Total score ranges from 0–18, with a higher score indicating worse HRQoL
	CfB in SF-36 PCS	• Standardised with a mean (SD) of 50 (10) in the general US population<br>• Higher scores reflect better health status
	CfB in BASMI	• Scores range from 0–13<br>• Higher scores indicate more severe limitation of movement
	CfB in MASES	• Measured in patients with MASES >0 at baseline<br>• Scores range from 0–13<br>• Higher scores indicate enthesitis at more sites
**Other pre-specified endpoints**	ASDAS MI	• Reduction of ≥2 points in the ASDAS relative to baseline visit
	ASDAS disease states	• Inactive disease (ID) – ASDAS <1.3<br>• Low disease activity (LDA) – ASDAS <2.1<br>• High disease activity (HDA) – ASDAS ≥2.1 to 3.5<br>• Very high disease activity (VHDA) – ASDAS >3.5
	BASDAI50	• 50% improvement in BASDAI
	CfB in PtGADA	• Measured on a numeric rating scale from 0–10<br>• Higher scores indicate higher activity of spondylitis

	CfB in morning stiffness	• Mean of BASDAI Q5&Q6
	Total resolution of enthesitis	• MASES=0 in subset of patients with MASES >0 at baseline
	CfB in TJC	• Scored from 0–44 in patients with TJC >0 at baseline<br>• Higher count indicates more tender joints
	CfB in SJC	• Scored from 0–44 in patients with SJC >0 at baseline<br>• Higher count indicates more swollen joints
	CfB in MRI SPARCC SIJ score	• Only reported for patients entering OLE from BE MOBILE 1 (nr-axSpA)<br>• Scored from 0–72<br>• Higher score indicates greater levels of inflammation in the SIJ
	CfB in MRI Berlin Spine score	• Only reported for patients entering OLE from BE MOBILE 2 (r-axSpA)<br>• Scored from 0–69<br>• Higher score indicates greater levels of inflammation in the spine

Abbreviations: ASDAS: Axial Spondyloarthritis Disease Activity Score; ASDAS HDA: ASDAS high disease activity; ASDAS LDA: ASDAS low disease activity; ASDAS MI: ASDAS major improvement; ASDAS vHDA: ASDAS very high disease activity; ASQoL: Ankylosing Spondylitis Quality of Life; BASDAI50: BASDAI 50% improvement; BASMI: Bath Ankylosing Spondylitis Metrology Index; CfB: change from baseline; HRQoL: health-related quality of life; MASES: Maastricht Ankylosing Spondylitis Enthesitis Index; MRI: magnetic resonance imaging; PtGADA: Patient's Global Assessment of Disease Activity; Q: question; SD: standard deviation; SF-36 PCS: Short-Form 36-item Health Survey Physical Component Summary; SPARCC: Spondyloarthritis Research Consortium of Canada; SIJ: sacroiliac joints SJC: swollen joint count; TJC: tender joint count.

Table S2. Fungal infections overview to Week 104

	Any BKZ 160 mg Q4W	
n (%) [EAIR/100 PY]	**Weeks 0–52 N=574[a]; 551.5 PY** (BE MOBILE 1&2)	**Weeks 52–104 N=518; 477.9 PY** (BE MOVING)
Any fungal infections	83 (14.5) [16.5]	53 (10.2) [11.8]
Candida infections	52 (9.1) [10.0]	31 (6.0) [6.8]
Oral candidiasis	42 (7.3) [8.0]	25 (4.8) [5.4]
Oropharyngeal candidiasis	5 (0.9) [0.9]	3 (0.6) [0.6]
Vulvovaginal candidiasis	4 (0.7) [0.7]	1 (0.2) [0.2]
Anal candidiasis	1 (0.2) [0.2]	0
Genital candidiasis	1 (0.2) [0.2]	0
Oesophageal candidiasis	1 (0.2) [0.2]	1 (0.2) [0.2]
Skin *Candida*	1 (0.2) [0.2]	0
Candida infection (unspecified)	0	2 (0.4) [0.4]
Fungal infections NEC	28 (4.9) [5.2]	22 (4.2) [4.7]
Vulvovaginal mycotic infection	10 (1.7) [1.8]	1 (0.2) [0.2]
Fungal skin infection	8 (1.4) [1.5]	8 (1.5) [1.7]
Oral fungal infection	6 (1.0) [1.1]	6 (1.2) [1.3]
Onychomycosis	4 (0.7) [0.7]	0
Fungal oesophagitis	1 (0.2) [0.2]	0
Oropharyngitis fungal	1 (0.2) [0.2]	0
Otitis externa fungal	1 (0.2) [0.2]	2 (0.4) [0.4]
Tongue fungal infection	1 (0.2) [0.2]	2 (0.4) [0.4]
Ear infection fungal	0	1 (0.2) [0.2]
Fungal infection (unspecified)	0	2 (0.4) [0.4]
Tinea infections	9 (1.6) [1.6]	6 (1.2) [1.3]
Tinea pedis	5 (0.9) [0.9]	2 (0.4) [0.4]
Tinea versicolour	2 (0.3) [0.4]	2 (0.4) [0.4]
Dermatophytosis of nail	1 (0.2) [0.2]	0
Tinea infection (unspecified)	1 (0.2) [0.2]	1 (0.2) [0.2]
Tinea capitis	0	1 (0.2) [0.2]

Safety set. Includes all data available up to the last Week 104 visit; if patients discontinued before Week 104, data are reported for up to 140 days from the last treatment dose, up to the data cut (July 2023). MedDRA (v19.0). [a]12 patients did not receive BKZ and are not included. Abbreviations: BKZ: bimekizumab; EAIR: exposure-adjusted incidence rate; NEC: not elsewhere classified; PY: patient-years; Q4W: every 4 weeks; TEAE: treatment-emergent adverse event.

Table S3. Efficacy outcomes to Week 104 (NRI and OC)

		BE MOVING Full Randomised Set N=586		
NRI:[a] n (%)		**Baseline**	**Week 52**	**Week 104**
**ASAS40**	NRI	-	347 (59.2)	304 (51.9)
**ASAS20**	NRI	-	429 (73.2)	381 (65.0)
**ASAS PR**	NRI	-	183 (31.2)	182 (31.1)
**ASAS 5/6**	NRI	-	334 (57.0)	286 (48.8)
**BASDAI50**	NRI	-	319 (54.4)	316 (53.9)
**Total resolution of enthesitis** (MASES=0)[b]	NRI	-	190 (49.4)	184 (47.8)
OC: Mean (SD), n		**Mean at baseline**	**CfB to Week 52**	**CfB to Week 104**
**ASDAS**	OC	3.7 (0.8), 586	−1.8 (1.0), 499	−1.9 (1.0), 449
**BASDAI**	OC	6.6 (1.3), 586	−3.8 (2.0), 515	−4.1 (2.1), 455
**BASFI**	OC	5.3 (2.2), 586	−2.8 (2.1), 516	−3.0 (2.3), 456
**BASMI**	OC	3.5 (1.6), 586	−0.7 (0.9), 496	−0.6 (1.0), 450
**MASES**[b]	OC	4.5 (3.1), 385	−3.2 (3.0), 328	−3.2 (3.0), 292
**Nocturnal spinal pain**	OC	6.7 (2.0), 586	−4.3 (2.5), 515	−4.5 (2.6), 456
**Morning stiffness** (mean of BASDAI Q5&Q6)	OC	6.8 (1.7), 586	−4.3 (2.3), 515	−4.6 (2.4), 455
**Fatigue** (BASDAI Q1)	OC	6.5 (1.6), 586	−3.2 (2.3), 515	−3.6 (2.3), 455
**ASQoL**	OC	9.1 (4.5), 586	−5.8 (4.6), 516	−6.0 (4.7), 456
**SF-36 PCS**	OC	34.0 (8.6), 586	12.2 (9.2), 516	13.0 (9.4), 456
**PtGADA**	OC	6.8 (1.9), 586	−3.9 (2.5), 516	−4.0 (2.7), 456
**TJC**[c]	OC	5.7 (6.1), 340	−4.0 (5.6), 294	−4.3 (5.8), 268
**SJC**[d]	OC	4.2 (4.3), 154	−3.5 (4.2), 133	−3.6 (4.1), 118

[a]Patients with intercurrent events were imputed as non-responders; [b]MASES score reported in patients with MASES>0 at baseline (n=385); [c]Assessed in patients with TJC>0 at baseline (n=340); [d]Assessed in patients with SJC >0 at baseline (n=154). Abbreviations: ASAS: Assessment of SpondyloArthritis international Society; ASAS20: ASAS 20% response; ASAS40: ASAS 40% response; ASAS 5/6: ASAS 5 out of 6 response criteria; ASAS PR: ASAS partial remission; ASDAS: Axial Spondyloarthritis Disease Activity Score; ASQoL: Ankylosing Spondylitis Quality of Life; BASDAI: Bath Ankylosing Spondylitis Disease Activity Index; BASDAI50: BASDAI 50% response; BASFI: Bath Ankylosing Spondylitis Functional Index; BASMI: Bath Ankylosing Spondylitis Metrology Index; CfB: change from baseline; MASES: Maastricht Ankylosing Spondylitis Enthesitis Score; NRI: non-responder imputation; OC: observed case; PtGADA: Patient's Global Assessment of Disease Activity; Q: question; SD: standard deviation; SF-36 PCS: Short-Form 36-Item Health Survey Physical Component Summary; SJC: swollen joint count; TJC: tender joint count.

Table S4. Efficacy outcomes to Week 104 (absolute values)

		BE MOVING Full Randomised Set N=586		
OC: Mean (SD), n<br>MI: Mean (SE)		**Baseline**	**Week 52**	**Week 104**
**ASDAS**	OC	3.7 (0.8), 586	1.9 (0.9), 499	1.8 (0.9), 449
	MI	3.7 (0.0)	2.0 (0.0)	1.9 (0.0)
**BASDAI**	OC	6.6 (1.3), 586	2.8 (2.0), 515	2.6 (2.1), 455
	MI	6.6 (0.1)	2.9 (0.1)	2.7 (0.1)
**BASFI**	OC	5.3 (2.2), 586	2.5 (2.1), 516	2.4 (2.3), 456
	MI	5.3 (0.1)	2.5 (0.1)	2.4 (0.1)
**BASMI**	OC	3.5 (1.6), 586	2.8 (1.5), 497	2.9 (1.5), 451
	MI	3.5 (0.1)	2.9 (0.1)	2.9 (0.1)
**MASES[a]**	OC	4.5 (3.1), 385	1.3 (2.2), 328	1.2 (2.1), 292
	MI	4.5 (0.2)	1.4 (0.1)	1.3 (0.1)
**Nocturnal spinal pain**	OC	6.7 (2.0), 586	2.4 (2.2), 515	2.3 (2.3), 456
	MI	6.7 (0.1)	2.5 (0.1)	2.4 (0.1)
**Morning stiffness**	OC	6.8 (1.7), 586	2.5 (2.1), 515	2.3 (2.2), 455
(mean of BASDAI Q5&Q6)	MI	6.8 (0.1)	2.6 (0.1)	2.5 (0.1)
**Fatigue** (BASDAI Q1)	OC	6.5 (1.6), 586	3.3 (2.3), 515	3.0 (2.2), 455
	MI	6.5 (0.1)	3.4 (0.1)	3.1 (0.1)
**ASQoL**	OC	9.1 (4.5), 586	3.3 (4.2), 516	3.2 (4.2), 456
	MI	9.1 (0.2)	3.5 (0.2)	3.5 (0.2)
**SF-36 PCS**	OC	34.0 (8.6), 586	46.2 (8.8), 516	46.8 (9.0), 456
	MI	34.0 (0.4)	46.0 (0.4)	46.5 (0.4)
**PtGADA**	OC	6.8 (1.9), 586	2.9 (2.2), 516	2.8 (2.4), 456
	MI	6.8 (0.1)	3.0 (0.1)	2.9 (0.1)
**TJC[b]**	OC	5.7 (6.1), 340	1.6 (3.7), 294	1.1 (3.0), 268
	MI	5.7 (0.3)	1.8 (0.2)	1.3 (0.2)
**SJC[c]**	OC	4.2 (4.3), 154	0.9 (3.1), 133	0.7 (2.7), 118
	MI	4.2 (0.4)	1.0 (0.3)	0.8 (0.2)

[a]MASES score reported in patients with MASES >0 at baseline (n=385); [b]Assessed in patients with TJC >0 at baseline (n=340); [c]Assessed in patients with SJC >0 at baseline (n=154). Abbreviations: ASDAS: Axial Spondyloarthritis Disease Activity Score; ASQoL: Ankylosing Spondylitis Quality of Life; BASDAI: Bath Ankylosing Spondylitis Disease Activity Index; BASFI: Bath Ankylosing Spondylitis Functional Index; BASMI: Bath Ankylosing Spondylitis Metrology Index; hs-CRP: high-sensitivity C-reactive protein; ID: inactive disease; LDA: low disease activity; MASES: Maastricht Ankylosing Spondylitis Enthesitis Score; MI: multiple imputation; NRI: non-responder imputation; OC: observed case; PtGADA: Patient's Global Assessment of Disease Activity; Q: question; SD: standard deviation; SE: standard error; SF-36 PCS: Short-Form 36-Item Health Survey Physical Component Summary; SJC: swollen joint count; TJC: tender joint count.

Table S5. Efficacy outcomes to Week 104, stratified by patients with nr-axSpA and r-axSpA (absolute values)

		BE MOVING Full Randomised Set N=586					
		BE MOBILE 1 (nr-axSpA) N=254			BE MOBILE 2 (r-axSpA) N=332		
NRI: n (%)<br>OC: n/N (%)<br>MI: Mean % (95% CI)		**Baseline**	**Week 52**	**Week 104**	**Baseline**	**Week 52**	**Week 104**
**ASAS40**	NRI	-	142 (55.9)	125 (49.2)	-	205 (61.7)	179 (53.9)
	OC	-	142/218 (65.1)	125/189 (66.1)	-	205/298 (68.8)	179/267 (67.0)
	MI	-	60.0 (53.8, 66.3)	58.9 (52.6, 65.2)	-	64.3 (59.1, 69.5)	61.0 (55.5, 66.5)
**ASAS20**	NRI	-	182 (71.7)	158 (62.2)	-	247 (74.4)	223 (67.2)
	OC	-	182/218 (83.5)	158/189 (83.6)	-	247/298 (82.9)	223/267 (83.5)
**ASAS PR**	NRI	-	76 (29.9)	78 (30.7)	-	107 (32.2)	104 (31.3)
	OC	-	76/216 (35.2)	78/189 (41.3)	-	107/298 (35.9)	104/266 (39.1)
**ASAS 5/6**	NRI	-	136 (53.5)	111 (43.7)	-	198 (59.6)	175 (52.7)
	OC	-	136/213 (63.8)	111/189 (58.7)	-	198/290 (68.3)	175/267 (65.5)
**ASDAS MI**	OC	-	84/211 (39.8)	81/187 (43.3)	-	120/288 (41.7)	126/262 (48.1)
	MI	-	35.0 (29.0, 40.9)	36.6 (30.4, 42.7)	-	38.5 (33.2, 43.7)	42.2 (36.8, 47.7)
**ASDAS LDA** (ASDAS <2.1)	OC	-	129/211 (61.1)	123/187 (65.8)	-	179/288 (62.2)	173/262 (66.0)
	MI	-	56.0 (49.7, 62.3)	58.8 (52.4, 65.2)	-	58.1 (52.7, 63.6)	60.4 (55.0, 65.9)
**ASDAS ID** (ASDAS <1.3)	OC	-	61/211 (28.9)	69/187 (36.9)	-	84/288 (29.2)	90/262 (34.4)
	MI	-	25.9 (20.4, 31.4)	31.0 (25.1, 36.9)	-	27.0 (22.1, 31.8)	30.3 (25.1, 35.4)
**BASDAI50**	NRI	-	131 (51.6)	129 (50.8)	-	188 (56.6)	187 (56.3)
	OC	-	131/217 (60.4)	129/189 (68.3)	-	188/298 (63.1)	187/266 (70.3)
**Total resolution of enthesitis[a]**	NRI	-	92 (49.5)	78 (41.9)	-	98 (49.2)	106 (53.3)
	OC	-	92/157 (58.6)	78/138 (56.5)	-	98/171 (57.3)	106/154 (68.8)
OC: Mean (SD), n<br>MI: Mean (SE)		**Baseline**	**Week 52**	**Week 104**	**Baseline**	**Week 52**	**Week 104**
**ASDAS**	OC	3.7 (0.7), 254	1.9 (0.9), 211	1.8 (0.9), 187	3.7 (0.8), 332	1.9 (0.8), 288	1.8 (0.9), 262
	MI	3.7 (0.1)	2.0 (0.1)	1.9 (0.1)	3.7 (0.0)	1.9 (0.1)	1.9 (0.1)

		BE MOVING Full Randomised Set<br>N=586					
		**BE MOBILE 1 (nr-axSpA)<br>N=254**			**BE MOBILE 2 (r-axSpA)<br>N=332**		
OC: Mean (SD), n<br>MI: Mean (SE)		**Baseline**	**Week 52**	**Week 104**	**Baseline**	**Week 52**	**Week 104**
**BASDAI**	OC	6.8 (1.3), 254	3.0 (2.1), 217	2.7 (2.1), 189	6.5 (1.3), 332	2.7 (1.9), 298	2.5 (2.0), 266
	MI	6.8 (0.1)	3.1 (0.1)	2.9 (0.1)	6.5 (0.1)	2.8 (0.1)	2.6 (0.1)
**BASFI**	OC	5.4 (2.3), 254	2.5 (2.2), 218	2.3 (2.2), 189	5.2 (2.1), 332	2.4 (2.0), 298	2.4 (2.3), 267
	MI	5.4 (0.1)	2.6 (0.1)	2.4 (0.2)	5.2 (0.1)	2.5 (0.1)	2.4 (0.1)
**BASMI**	OC	3.0 (1.3), 254	2.4 (1.3), 212	2.4 (1.2), 188	3.9 (1.6), 332	3.1 (1.6), 285	3.2 (1.7), 263
	MI	3.0 (0.1)	2.5 (0.1)	2.4 (0.1)	3.9 (0.1)	3.2 (0.1)	3.2 (0.1)
**MASES**[a]	OC	4.8 (3.3), 186	1.4 (2.4), 157	1.4 (2.2), 138	4.3 (2.8), 199	1.2 (2.0), 171	1.0 (2.0), 154
	MI	4.8 (0.2)	1.6 (0.2)	1.6 (0.2)	4.3 (0.2)	1.3 (0.1)	1.0 (0.1)
**Nocturnal spinal pain**	OC	6.8 (2.1), 254	2.5 (2.3), 217	2.3 (2.4), 189	6.7 (1.9), 332	2.3 (2.1), 298	2.3 (2.2), 267
	MI	6.8 (0.1)	2.6 (0.2)	2.5 (0.2)	6.7 (0.1)	2.4 (0.1)	2.4 (0.1)
**Morning stiffness** (mean BASDAI Q5&Q6)	OC	6.9 (1.7), 254	2.5 (2.1), 217	2.3 (2.1), 189	6.7 (1.8), 332	2.6 (2.1), 298	2.3 (2.2), 266
	MI	6.9 (0.1)	2.6 (0.1)	2.4 (0.1)	6.7 (0.1)	2.6 (0.1)	2.5 (0.1)
**Fatigue** (BASDAI Q1)	OC	6.6 (1.7), 254	3.5 (2.4), 217	3.2 (2.4), 189	6.4 (1.5), 332	3.2 (2.2), 298	2.8 (2.1), 266
	MI	6.6 (0.1)	3.6 (0.2)	3.3 (0.2)	6.4 (0.1)	3.2 (0.1)	2.9 (0.1)
**ASQoL**	OC	9.4 (4.5), 254	3.6 (4.2), 218	3.3 (4.3), 189	8.9 (4.6), 332	3.1 (4.1), 298	3.1 (4.2), 267
	MI	9.4 (0.3)	3.8 (0.3)	3.8 (0.3)	8.9 (0.3)	3.2 (0.2)	3.2 (0.2)
**SF-36 PCS**	OC	33.4 (8.5), 254	45.6 (9.1), 218	46.5 (9.2), 189	34.4 (8.6), 332	46.7 (8.6), 298	47.0 (8.8), 267
	MI	33.4 (0.5)	45.2 (0.6)	45.9 (0.6)	34.5 (0.5)	46.7 (0.5)	46.9 (0.5)
**PtGADA**	OC	7.0 (1.9), 254	2.9 (2.3), 218	2.9 (2.5), 189	6.7 (1.9), 332	2.8 (2.2), 298	2.7 (2.3), 267
	MI	7.0 (0.1)	3.1 (0.2)	3.1 (0.2)	6.7 (0.1)	2.9 (0.1)	2.8 (0.1)
**TJC**[b]	OC	6.1 (6.3), 163	2.2 (4.7), 138	1.6 (3.9), 123	5.4 (5.9), 177	1.1 (2.5), 156	0.7 (1.9), 145
	MI	6.1 (0.5)	2.4 (0.4)	1.8 (0.3)	5.4 (0.4)	1.3 (0.2)	0.9 (0.2)
**SJC**[c]	OC	4.0 (4.7), 88	1.2 (4.1), 71	0.8 (3.3), 64	4.5 (3.9), 66	0.5 (1.2), 62	0.5 (1.6), 54
	MI	4.0 (0.5)	1.2 (0.4)	0.9 (0.3)	4.5 (0.5)	0.7 (0.2)	0.6 (0.2)

[a]MASES score reported in patients with MASES >0 at baseline (nr-axSpA: n=186; r-axSpA: 199); [b]Assessed in patients with TJC >0 at baseline (nr-axSpA: n=163; r-axSpA: n=177); [c]Assessed in patients with SJC >0 at baseline (nr-axSpA: n=88; r-axSpA: n=66). Abbreviations: ASAS: Assessment of SpondyloArthritis international Society; ASAS20: ASAS 20% response; ASAS40: ASAS 40% response; ASAS 5/6: ASAS 5 out of 6 response criteria; ASAS PR: ASAS partial remission; ASDAS: Axial Spondyloarthritis Disease Activity Score; ASDAS ID: ASDAS inactive disease; ASDAS LDA: ASDAS low disease activity; ASDAS MI: ASDAS major improvement; ASQoL: Ankylosing Spondylitis Quality of

Life; BASDAI: Bath Ankylosing Spondylitis Disease Activity Index; BASDAI50: BASDAI 50% response; BASFI: Bath Ankylosing Spondylitis Functional Index; BASMI: Bath Ankylosing Spondylitis Metrology Index; MASES: Maastricht Ankylosing Spondylitis Enthesitis Score; MI: multiple imputation; NRI: non-responder imputation; OC: observed case; PtGADA: Patient's Global Assessment of Disease Activity; Q: question; SD: standard deviation; SE: standard error; SF-36 PCS: Short-Form 36-Item Health Survey Physical Component Summary; SJC: swollen joint count; TJC: tender joint count.

Figure S1. Study design

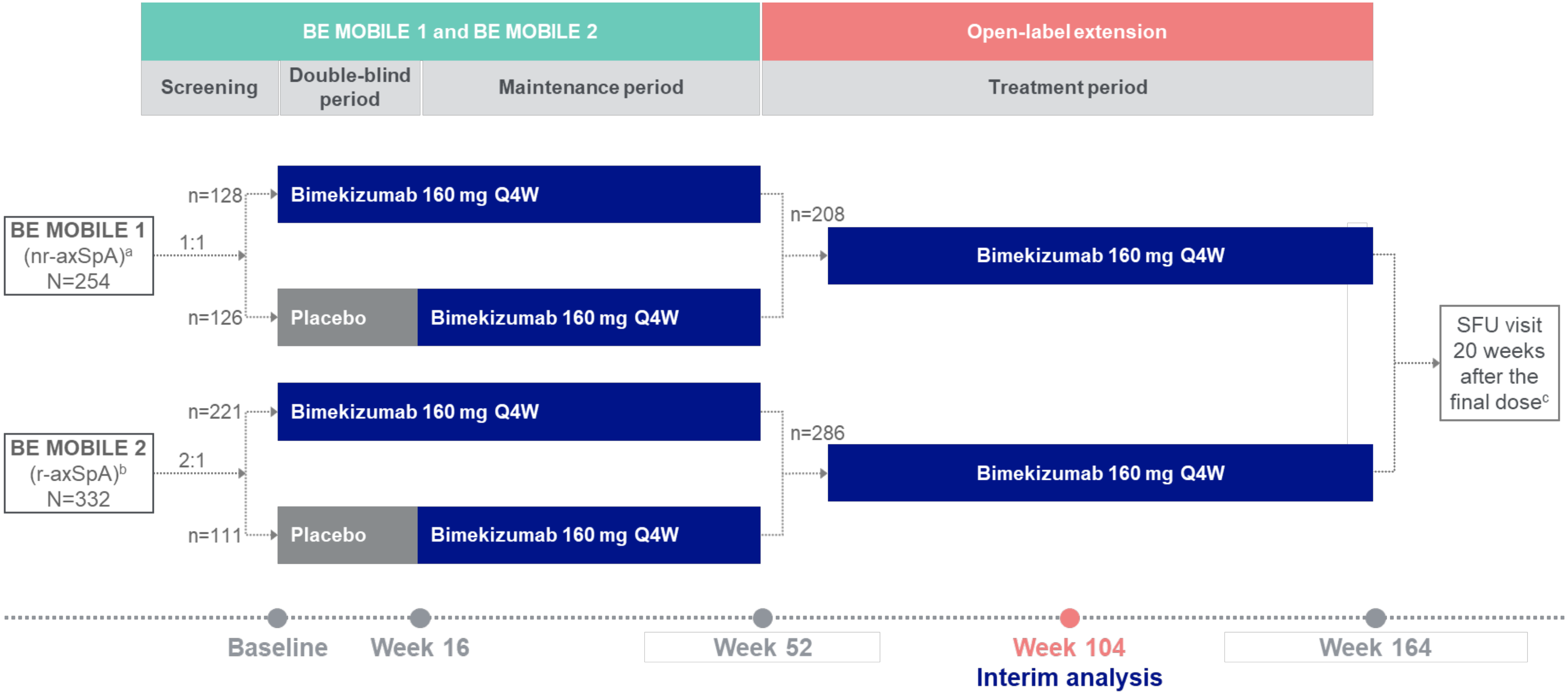

[a]Included patients had adult-onset nr-axSpA fulfilling ASAS classification criteria and objective signs of inflammation (active sacroiliitis on MRI and/or elevated CRP [≥6 mg/L]); [b]Included patients had radiographic evidence of r-axSpA fulfilling modified New York criteria; [c]Study participants will receive their final treatment dose at Week 108; the SFU Visit will be conducted 20 weeks after the final treatment dose. OLE: open-label extension; Q4W: every 4 weeks; SFU: safety follow-up.

Figure S2. Patient disposition from baseline to Week 104

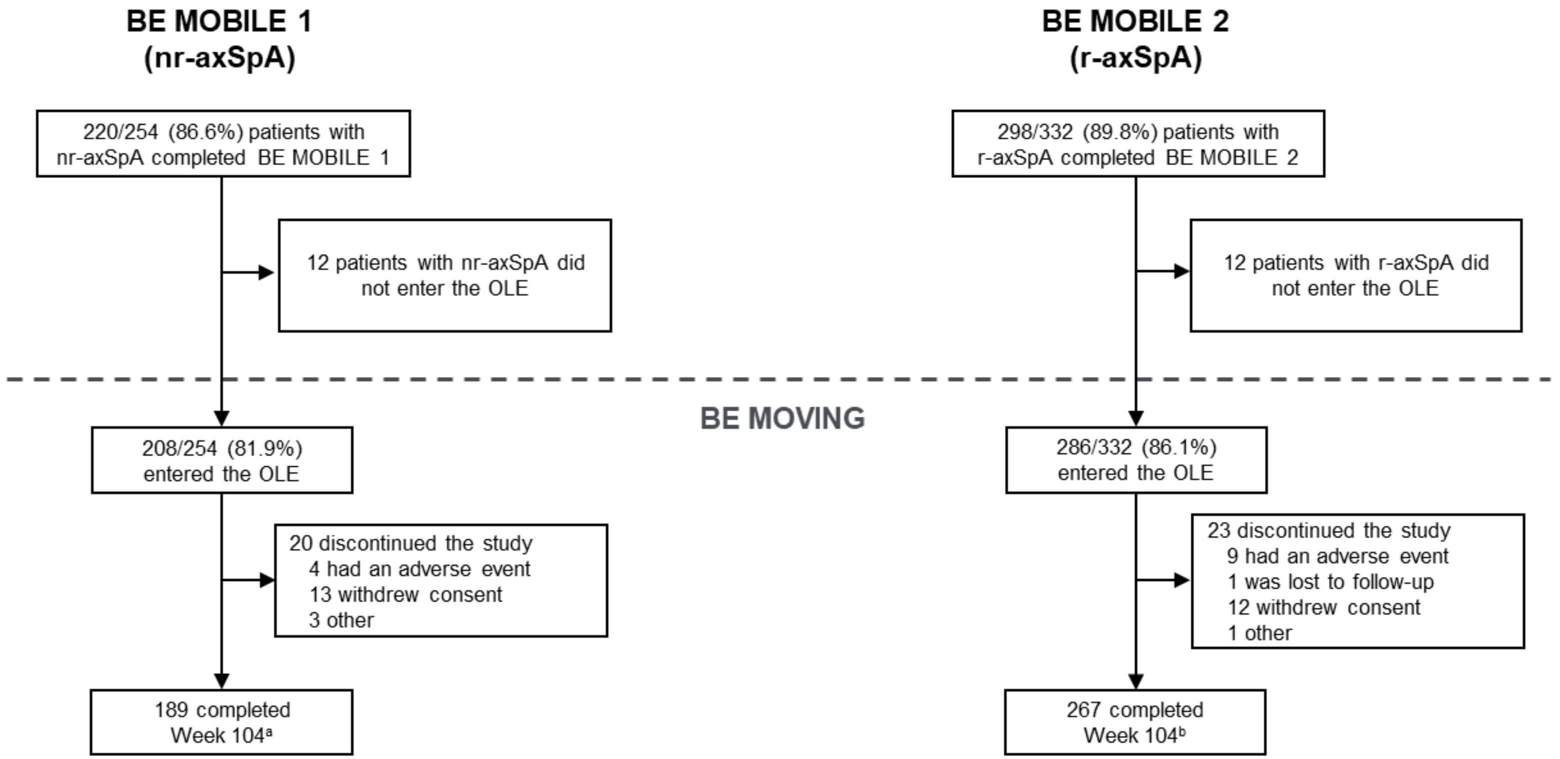

Ongoing study; patient discontinuation reported as per the interim data cut in July 2023. Patients may have discontinued after Week 104 but before the interim data cut. [a]188 patients originally randomised in BE MOBILE 1 were still active in the study at the time of the interim data cut in July 2023; [b]263 patients originally randomised in BE MOBILE 2 were still active in the study at the time of the interim data cut in July 2023. Abbreviations: nr-axSpA: non-radiographic axial spondyloarthritis; OLE: open-label extension; r-axSpA: radiographic axial spondyloarthritis.
